# Alterations in the “Gut–Liver Axis” on Rats with Immunological Hepatic Fibrosis

**DOI:** 10.1155/2023/5577850

**Published:** 2023-09-21

**Authors:** Zhaoyao Qi, Xinxin Qi, Yuanhui Xu, Hongguang Sun, Dengfeng Li, Jincun Liu, Meili Cong, Tao Liu

**Affiliations:** School of Public Health, Xinjiang Medical University, No. 393 Xinyi Road, Urumqi 830011, Xinjiang Uyghur Autonomous Region, China

## Abstract

There remains a lack of standard models that have all the characteristics of human diseases. Especially in immunological hepatic fibrosis, the bovine serum albumin (BSA)-induced liver fibrosis models have the same developmental mechanisms as human liver fibrosis models, but have received little attention. We standardized a BSA-induced liver fibrosis model in rats and thoroughly assessed its pathological characteristics. We also used 16S sequencing to assess homeostasis of the intestinal microflora of rats with BSA-induced liver fibrosis and detected various differential metabolites in the serum of these rats using ultrahigh-performance liquid chromatography–tandem mass spectrometry (UHPLC-MS/MS). We observed stable and unambiguous histological changes in liver tissue morphology and remarkably high concentrations of inflammatory markers in the serum of BSA-induced liver fibrosis rats. In keeping with the fact that BSA induction can cause gut microbiota disorders in rats. UHPLC-MS/MS analysis of rat serum samples in positive-ion mode and negative-ion mode revealed 17 and 25 differential metabolites, respectively. Network analysis revealed that phenylalanine or tyrosine metabolites (e.g., PAGln) were the predominant metabolites in the sera of BSA-induced liver fibrosis rats. Taken together, our results suggest that disorders of amino acid metabolism caused by the gut microbiota may play an important role in the progression of immunological hepatic fibrosis.

## 1. Introduction

Liver disease is a major cause of morbidity and mortality and is also a substantial economic burden. Worldwide each year, more than 300 million people are infected with hepatitis B or C (HBV/HCV) and approximately 1.4 million die from HBV/HCV [1–3]. Liver fibrosis is a wound-healing response to long-term liver injury, such as that caused by infection with HBV/HCV or by toxins, drugs, or cholestasis [4, 5]. Key events in liver fibrosis are the activation of hepatic stellate cells (HSCs) and the massive accumulation of extracellular matrix (ECM) proteins [5]. It is known that fibrosis includes an oxidative damage, inflammatory, and immune response and HSCs and their activation in hepatocytes [6]. Moreover, the development and progression of liver fibrosis in involved in liver disease and associated with the gut–liver axis [7–10].

Intestine and the liver are connected in anatomy, physiology, and function, and about 70% of the blood flow of liver is provided by intestine through portal vein, thus transmitting information from intestine to the liver [11, 12]. Moreover, the liver transmits information to the gut in the form of bile acids. Thus, there are metabolic connections between the gut and liver. Moreover, the gut microbiota, gut epithelium, and intestinal mucosal immune system are interdependent and form a complex interacting network of components that constitute the intestinal microecology [13], which plays a crucial role in the development and progression of liver fibrosis, which primarily involves the gut microbiota and serum metabolism [14–16]. Also, the mediators and cytokines in inflammation play an important role in fibrosis [5], such as high levels of plasma ammonia and inflammation are related to changes of blood flow velocity in organs in patients with liver cirrhosis [17].

Despite the bovine serum albumin (BSA)-induced liver fibrosis model being an excellent immunological hepatic fibrosis model, it has not received much attention from the researchers worldwide. The model is created by administering multiple BSA injections to an animal to generate immune complex (IC)-mediated liver fibrosis, which is similar to the metabolic process involved in the clinical development and progression of fibrogenesis [18–20]. Herein, we detected changes in gut microbiota and serum metabolism in BSA-induced liver fibrosis rats, and explored the possible roles of these changes in the progression of liver fibrosis. Our findings may support the application of the BSA model in microecological multiomics studies.

## 2. Methods

### 2.1. Animals

Forty-three healthy adult-specific-pathogen-free Sprague-Dawley rats (male; 220–270 g) were purchased from the center of animal experiments of the Xinjiang Medical University, China (Grant Number SCXK (xin) 2018-0002). All the rats were housed under controlled environmental conditions (22°C–25°C and a 12-hr light/dark cycle) and had free access to food (standard rat pellets) and water (Grant Number SYXK (xin) 2018-0003). All the experiments were conducted in strict compliance with the animal welfare and ethical principles of the center of animal experiments of the Xinjiang Medical University and approved by the Ethics Committee of the Animal Experimental Center of the center of animal experiments of the Xinjiang Medical University (confirmation number: IACUC-20220127-4).

### 2.2. Establishment of the BSA-Induced Liver Fibrosis Rat Model

**Figure 1:**
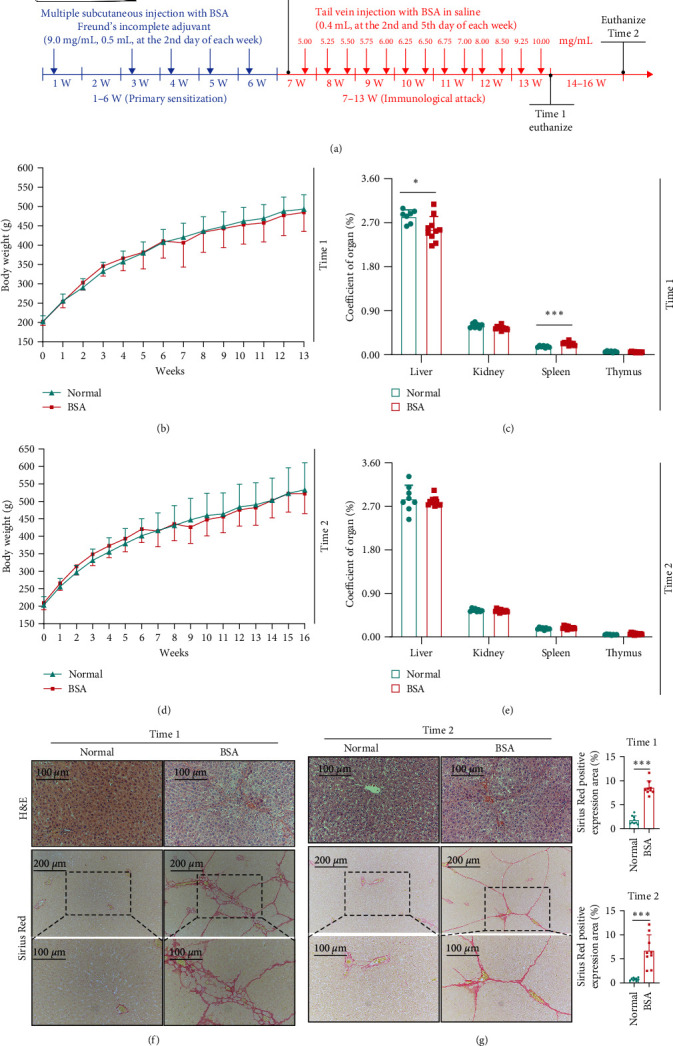
Detailed scheme of the establishment of the BSA model (a). Body weight profiles for euthanasia at times 1 and 2 ((b) and (d)). Coefficients for the liver, spleen, kidney, and thymus were calculated as follows ((c) and (e)). Histologic analysis of liver tissues at times 1 and 2 ((f) and (g)). Results compared with the normal group;  ^*∗*^*P* < 0.05 and  ^*∗∗∗*^*P* < 0.001.

After adaptive feeding for 3 days, the rats were randomly allocated to the normal group or BSA group. Rats in the BSA group were treated with BSA (Sigma, USA, Cat: A1933-5G) to induce liver fibrosis (Figure 1(a)), whereas rats in the normal group were treated with the same volume of saline.

Phase 1 of primary sensitization (1–6 weeks): On the second day of each week, the BSA group was administered multiple subcutaneous injections of BSA Freund's incomplete adjuvant (intradermally in the cervical back and lateral extremities; 0.5 mL (9 mg/mL)). Almost 7 days after the fifth injection, blood was extracted from the retinal capillary plexus and used for the detection of serum anti-BSA antibodies using the Agar-gel double diffusion test.

Phase 2 of immunological attack (7–13 weeks): On days 2 and 5 of each week, 0.4 mL of BSA in saline was injected into the tail vein of rats in the BSA group (including those negative for anti-BSA antibody) to observe pathological changes in these animals.

### 2.3. Animal Samples

The rats were fasted for 12 hr and then anesthetized with 20% urethane (0.2 mL/100 g). Blood was then extracted from the abdominal aorta, and the serum was obtained and stored at −80°C refrigerator until further use. The liver tissue was then rapidly thawed and fixed in 4% paraformaldehyde. The cecal and colon contents were collected, merged them into the intestinal contents, frozen in liquid nitrogen, and stored at −80°C until the intestinal flora was sequenced. The wet weights and weights of the liver, spleen, kidney, and thymus were recorded at the time of dissection. Organ coefficient = organ mass/(body weight/100) × 100%.

### 2.4. Enzyme-Linked Immunosorbent Assay (ELISA)

ELISA was conducted using kits [21], according to the manufacturer's instructions. An interleukin (IL)-1*β* kit (China, Cat: E-EL-R0012c), a rat tumor necrosis factor alpha (TNF-*α*) ELISA kit (China, Cat: E-EL-R2856c), and an IL-6 ELISA kit (China, Cat: E-EL-R0015c) were purchased from Elabscience Biotechnology Co. Ltd. China. A Type-IV Collagen (Col-IV) ELISA kit (China, Cat: D731044-96T) was purchased from Shanghai Sangon Biotech Co. Ltd. and a Laminin (LN) ELISA kit (China, Cat: CSB-E04646r) was purchased from Cusbio.

### 2.5. Histological Analysis of Liver Tissues

Liver tissues were fixed in buffered 4% paraformaldehyde, dehydrated, paraffin-embedded, sliced, and then placed onto slides. These were subjected to hematoxylin–eosin (H&E) staining and Sirius Red coloration and then observed under a light microscope (×100 or ×200) to observe pathological changes in liver tissue. The distribution and expression of Col-I, Col-III, and *α*-smooth muscle actin (*α*-SMA) in liver tissues were detected through immunohistochemical analyses using collagen I rabbit polyclonal antibody (Servicebio, China, Cat: GB11022-3), collagen III rabbit polyclonal antibody (Servicebio, China, Cat: GB111629), and *α*-SMA rabbit polyclonal antibody (Servicebio, China, Cat: GB111364).

### 2.6. Biochemical Assays

Liver function indicators, such as serum alanine transaminase (ALT), aspartate aminotransferase (AST), albumin (ALB), total bilirubin (TBIL), total protein (TP), and serum LN, were detected using an automatic biochemical analyzer (Mind-ray, BS-240 Vet), and Col-IV, TNF-*α*, IL-6, and IL-1*β* were detected using ELISA. Serum ALT, AST, TBIL, TP, and ALB kits were purchased from Xin'ao Biotechnology Business Department Co. Ltd. China.

### 2.7. Bacterial DNA Extraction and 16S Sequencing

Total genomic DNA was extracted from samples using the cetyltrimethylammonium bromide method. The 16S rRNA genes of distinct regions (V3–V4) were amplified using specific barcoded primers (341 F (5′-CCTAYGGGRBGCASCAG-3′) and 806R (5′-GGACTACNNGGGTATCTAAT-3′)).

### 2.8. Untargeted Metabolomics

Ultrahigh-performance liquid chromatography–tandem mass spectrometry (UHPLC-MS/MS) was performed on a Vanquish UHPLC system (Thermo Fisher, Germany) coupled with an Orbitrap Q-Exactive^TM^ HF-X mass spectrometer (Thermo Fisher). The samples were separated on a Hypesil Gold column (100 × 2.1 mm, 1.9 *μ*m) using a 12-min linear gradient at a flow rate of 0.2 mL/min. Eluents A and B for positive-ion mode were 0.1% formic acid in water and methanol, respectively, whereas eluents A and B for negative-ion mode were 5 mM ammonium acetate (pH 9.0) and methanol, respectively. The solvent gradient was as follows: 2% B, 1.5 min; 2%–85% B, 3 min; 85%–100% B, 10 min; 100%–2% B, 10.1 min; and 2% B, 12 min. The spectrometer was operated in positive- and negative-ion mode, respectively, with a spray voltage of 3.5 kV, a capillary temperature of 320°C, a sheath gas flow rate of 35 psi, an auxiliary gas flow rate of 10 L/min, an S-lens radio frequency of 60, and an auxiliary gas temperature of 350°C.

### 2.9. Sequencing and Metabolites Data Analysis

The *α* diversity index set contains four indexes: Chao1, Faith PD, Shannon, and observed features. Principal coordinate analysis (PCoA) was used to indicate the *β* diversity of the data via the weighted-UniFrac algorithm. An analysis of similarities test was used to test the significance of differences between the BSA and normal groups. Linear discriminant analysis (LDA) effect size (LEfSe) was used to detect species whose relative abundances differed significantly (biomarker species) between the BSA and normal groups (Kruskal–Wallis test). The differential abundance of amplicon sequence variants (ASVs) was using DESeq2 analysis (the R package DESeq2). Characteristic species determined by the Kruskal–Wallis test at the genus level. A Spearman rank correlation was determined to identify the significant gut microbiota species and environmental factors. The relationship between community changes in the significant species and different classification levels and the environmental factors was examined using the Mantel test in R package inkET (version 0.0.3). Pathway enrichment analysis of selected annotated differential species performed using STAMP 2.1.3 [22] and the Kyoto Encyclopedia of Genes and Genomes (KEGG) database (https://www.genome.jp/kegg/pathway.html). The significant species (LDA score > 2) were assessed using the LDA and are displayed in a histogram of the LDA value distribution.

The metabolic differences identified through orthogonal projections to the latent structure-discriminant analysis model were studied by choosing the variable importance in projection (VIP) as the defining variable (VIP > 1), and a metabolite variable with a *t*-test *P* < 0.05 and a fold change of >1 were used to determine the metabolic characteristics of the groups. SIMCA 14.2 was used to screen the related differential metabolites using single dimensional and multidimensional methods. These metabolites were annotated using the KEGG database and Human Metabolome Database (http://hmdb.ca/metabolites). Spearman rank correlation were used network analyse of serum differential metabolites and significant gut microbiota species.

### 2.10. Statistical Analysis

All the data are presented as means ± standard errors of the mean. The data were analyzed using SPSS 24.0. The two data groups were subjected to a normality test and a homogeneity of variance test. If the assumption for homogeneity of variance was met, we analyzed the data using the Student's *t*-test; otherwise, we used the Wilcoxon rank-sum test. *P* < 0.05 was considered statistically significant.

## 3. Results

### 3.1. Evaluation of the BSA-Induced Liver Fibrosis Rat Model

Figure 1(a) depicts the entire experimental duration. The animal mortality rate in the process of the model establishment was 17.86%, whereas the success rate for the model establishment was 100% (*Supplementary 1*). Throughout the experiment, compared with the normal group, there were no significant differences in the body weights of the BSA group (Figures 1(b) and 1(d)). Compared with the normal group at time 1, in the BSA group the liver coefficient decreased significantly (*P* < 0.05), but the spleen coefficient increased considerably (*P* < 0.001) (Figure 1(c)). There were no significant differences at time 2 (Figure 1(e)).

**Figure 2:**
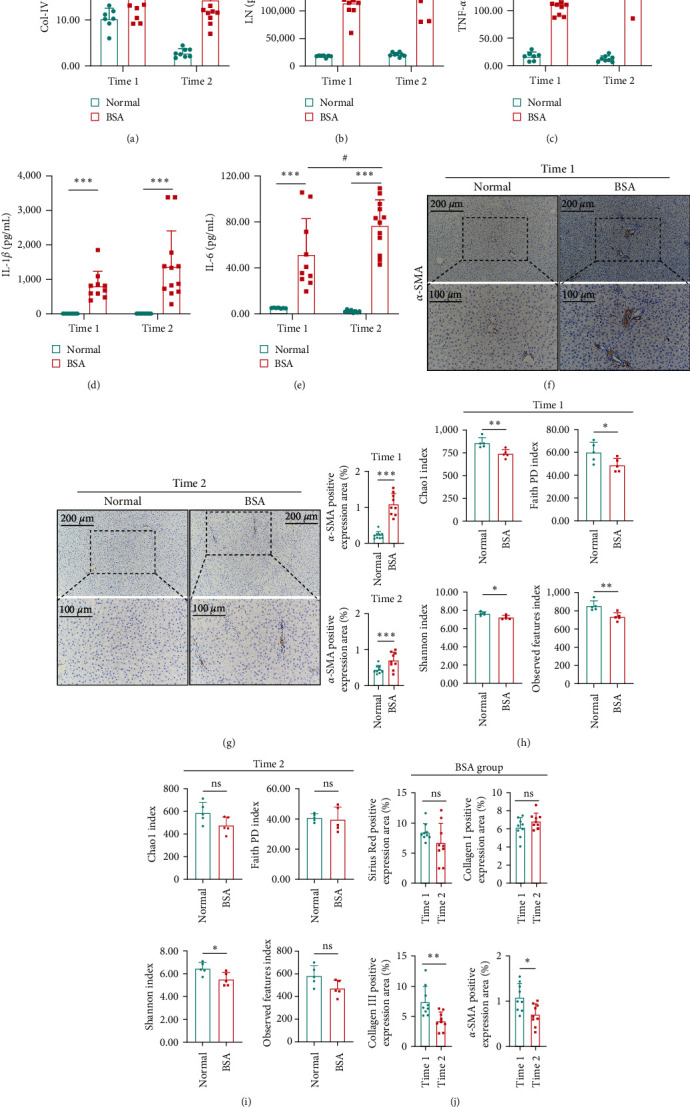
Data of serum Col-IV (a), LN (b), and inflammatory factors ((c), (d), and (e)). Results compared with the normal group;  ^*∗*^*P* < 0.05,  ^*∗∗*^*P* < 0.01, and  ^*∗∗∗*^*P* < 0.001. Results compared with time 1, #*P* < 0.05 and ###*P* < 0.001. The distribution and expression of *α*-SMA in the liver tissue were detected by immunohistochemistry ((f) and (g)). The *α* diversity index ((h) and (i)). In BSA group, the pathological scores of hepatic fibrosis were compared at time 1 and time 2 (j). The Sirius Red staining and IHC evaluation (Type I, III collagen and *α*-SMA) have beeen provided (j). NS indicates nonsignificance.

The livers of the BSA group rats showed significant fibrosis. At time 1 (Figure 1(f)), and time 2 (Figure 1(g)), the pathological sections of the livers stained with H&E and Sirius Red indicated that the livers of the BSA group rats had significant deposits of ECM. Moreover, immunohistochemistry of the liver tissues from time 1 and time 2 indicated that hepatic Col-I (*Supplementary 2*) and Col-III (*Supplementary 2*) expression were significantly increased in the BSA group (*P* < 0.001). Moreover, *α*-SMA expression was significantly increased in the BSA group, primarily near the portal vein (Figures 2(f) and 2(g)) (*P* < 0.001). In addition, the expressions of Col-III (*P* < 0.01) and *α*-SMA (*P* < 0.05) in the liver of BSA group at time 2 were significantly increased compared with those of BSA group at time 1. However, there was no statistical difference between Col-I and Sirius Red staining (Figure 2(j)). These pathological changes were not observed in the normal group. In addition, at time 2, the expression of both Col-IV (Figure 2(a)) and LN (Figure 2(b)) were significantly increased in the serum of the BSA group compared with in the serum of the normal group (*P* < 0.001).

There were few changes in the liver function indexes in the BSA group (*Supplementary 2*); with only the expression of TBIL and AST/ALT increasing, at time 2, compared with the normal group (*P* < 0.01) (*Supplementary 2*). However, there was an obvious inflammatory response in the BSA group: compared with the normal group, the levels of certain inflammatory factors, including TNF-*α* (Figure 2(c)), IL-1*β* (Figure 2(d)), and IL-6 (Figure 2(e)), were significantly increased in the BSA group (*P* < 0.001).

### 3.2. Structural and Compositional Differences in the Gut Microbiome after BSA Induction

At time 1, although the liver pathology and gut microbiome were significantly changed in the BSA group, it was difficult to identify whether the changes were directly induced by BSA or caused by BSA-induced liver fibrosis. Eight hundred forty-seven ASVs were specific to the BSA group and nine hundred and ninety-three ASVs were shared by the BSA and normal groups (*Supplementary 2*). Compared with those for the normal group, the *α* diversity indexes (Chao1, Faith PD, Shannon, and observed features at time 1) for the BSA group decreased (Figure 2(h)). The species distribution of the results was conducted at the genus level (*Supplementary 2*). PCoA indicated that the gut microbiota of the BSA group was significantly altered compared with that of the normal group at time 1 (*R* = 0.316, *P* < 0.05) (*Supplementary 2*).

**Figure 3:**
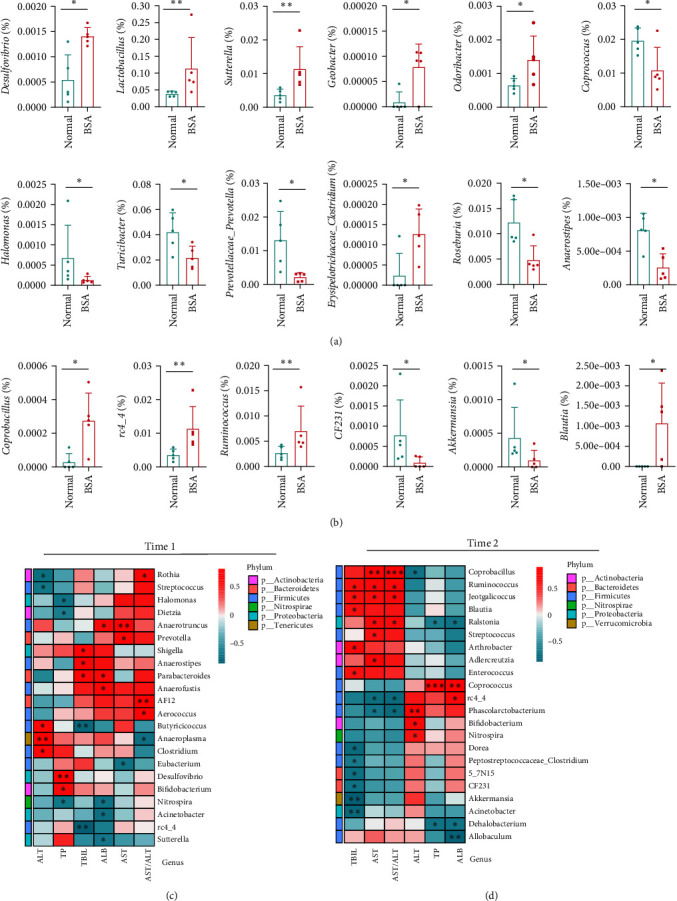
Characteristic species at times 1 (a) and 2 (b) determined by the Kruskal–Wallis test at the genus level. A Spearman rank correlation was determined to assess the correlations between the gut microbiota species and indicators of liver function ((c) and (d)).  ^*∗*^*P* < 0.05,  ^*∗∗*^*P* < 0.01, and  ^*∗∗∗*^*P* < 0.001.

Compared with the normal group, six differential species were found to have decreased and six differential species were found to have significantly increased in the BSA group (*P* < 0.05) (Figure 3(a)). The gut microbiota with increased relative abundance were primarily members of the *Proteobacteria* and *Bacteroidetes* phyla, whereas those with decreased relative abundance were primarily members of the *Proteobacteria* and *Firmicutes* phyla (*Supplementary 2*). The DESeq2 volcano plot of the BSA group showed (compared with the normal group) decreases in the relative abundances of *Prevotellaceae_Prevotella*, *Corynebacterium*, *Halomonas*, and *Bacillus* and increases in the relative abundances of *Akkermansia* (*Supplementary 2*) (Supplementary data of DESeq2 analysis, *Supplementary 1*). A Kruskal–Wallis test of the BSA group compared with the normal group showed increases and decreases in the relative abundances of characteristic species (Figure 3(a)) (Supplementary data of Kruskal–Wallis test, *Supplementary 1*). LEfSe identified characteristic species in the BSA and normal group (*Supplementary 2*).

### 3.3. Correlations between the Gut Microbiome and BSA-Induced Changes

**Figure 4:**
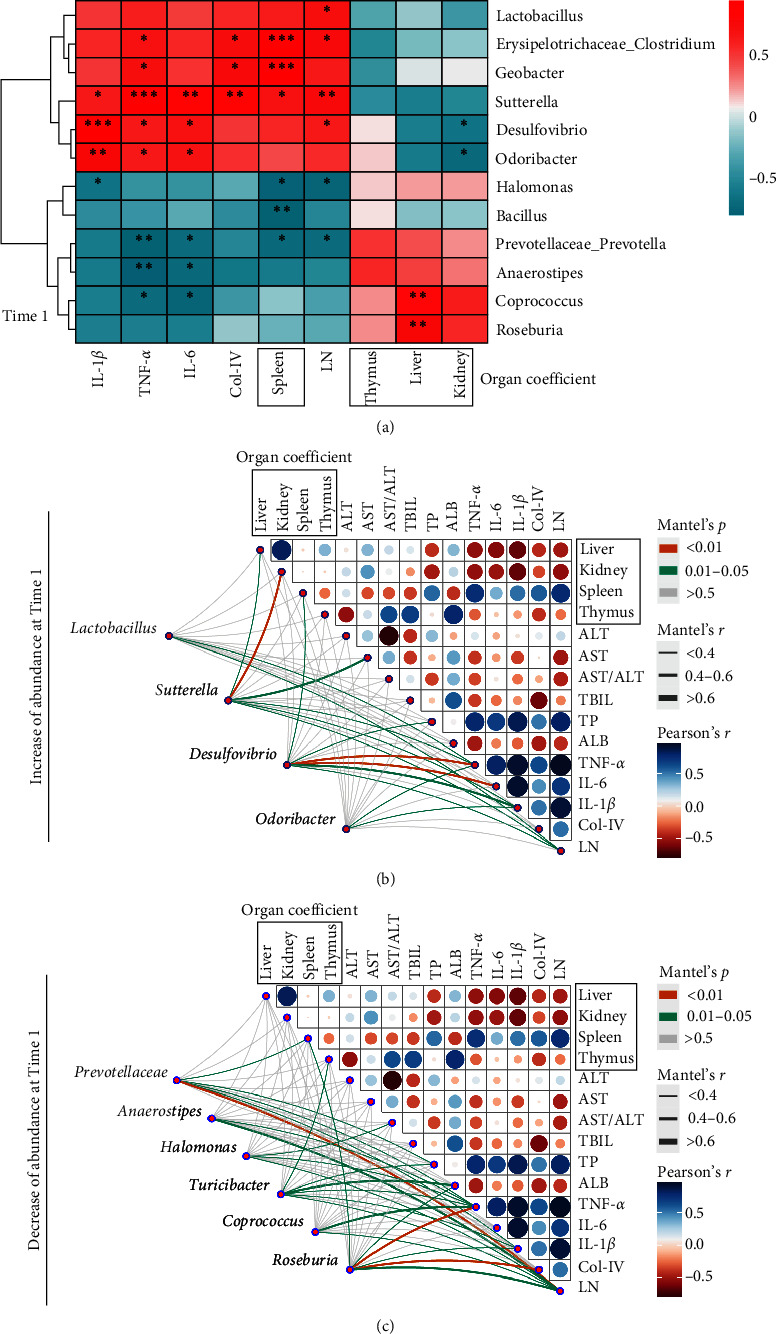
A Spearman rank correlation (a). Results compared with the normal group;  ^*∗*^*P* < 0.05,  ^*∗∗*^*P* < 0.01, and  ^*∗∗∗*^*P* < 0.001. The Mantel test ((b) and (c)).

At time 1, Mantel test results showed that *Sutterella* and *Desulfovibrio* were more closely related to other experimental indicators (i.e., TNF-*α*, IL-1*β*, IL-6, Col-IV, and LN) among the differential species with increased relative abundances in the BSA group (Figure 4(b)). However, *Prevotellaceae*, *Halomonas*, *Turicibacter*, and *Roseburia* were more closely associated with environmental factors in the species with decreased relative abundance in the BSA group (Figure 4(c)).

No significant between-group differences were found in the liver function indices at time 1. However, a significantly positive correlation was found between the abundance of *Desulfovibrio* and TP, whereas a negative correlation was found between the abundance of *Halomonas* and TP (*P* < 0.05) (Figure 3(c)). A significantly positive correlation was also found between the abundances of *Lactobacillus*, *Erysipelotrichaceae_Clostridium*, *Geobacter*, *Sutterella*, *Desulfovibrio*, and *Odoribacter* and inflammatory factors (TNF-*α*, IL-1*β*, and IL-6), LN, Col-IV, and the spleen coefficient. In contrast, a significantly negative correlation was found between the abundances of *Halomonas*, *Bacillus*, *Prevotellaceae_Prevotella*, *Anaerostipes*, *Coprococcus*, and *Roseburia* and the above-mentioned indicators (*P* < 0.05) (Figure 4(a)). In addition, a significantly positive correlation was noted between the abundances of *Coprococcus* and *Roseburia* and the liver coefficient (*P* < 0.05) (Figure 4(a)).

### 3.4. Evaluation of the Predictive Performance of the Gut Microbiome after BSA Induction

In terms of KEGG level 2 functions: compared with the normal group, in the gut microbiota of the BSA group at time 1, the expression of genes associated with cell motility and the immune system were downregulated and the expression of genes associated with endocrine and metabolic disease and with the excretory system were upregulated (*Supplementary 2*).

**Figure 5:**
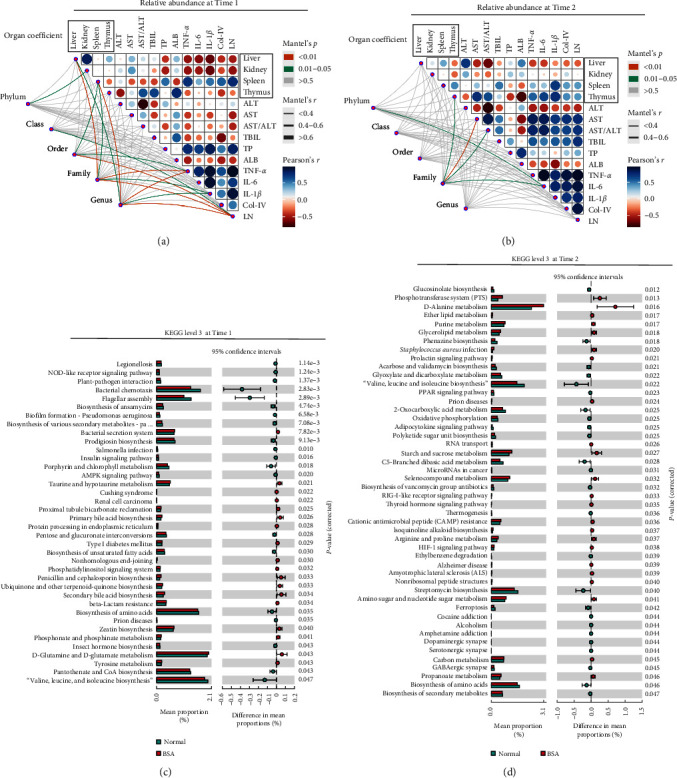
The Mantel test (a and b). Pathway enrichment analysis (level 3) of the selected annotated differential species at times 1 (c) and 2 (d).

In terms of KEGG level 3 functions: in the gut microbiota of the BSA group at time 1, the expression of genes associated with the expression of genes associated with the bacterial secretion system, primary bile acid biosynthesis, D-glutamine and d-glutamate metabolism, and tyrosine metabolism were upregulated (Figure 5(c)).

### 3.5. Structural and Compositional Differences in the Gut Microbiome of BSA-Induced Liver Fibrosis Rats

Based on the Mantel test results, the abundances of characteristic microbiota were decreased at time 2 (Figure 5(b)) compared with time 1 (Figure 5(a)). The Venn diagram shows that there were 763 ASVs specific to the BSA group and 635 ASVs present in the BSA and normal groups (*Supplementary 2*). Compared with time 1, the number of specific and common ASVs decreased at time 2 in the BSA group (*Supplementary 2*). Compared with the normal group, only the Shannon index for the BSA group decreased at time 2 (Figure 2(i)), and it exhibited no significant reductions in the Chao1 and Faith PD indexes or observed features. The species distribution was described at the genus level in the BSA and normal group (*Supplementary 2*). The PCoA results revealed no significant between-group differences at time 2 (*R* = 0.200, *P*=0.128) (*Supplementary 2*).

Compared with the normal group, in the BSA group, the abundances of two differential species decreased and the abundances of four differential species significantly increased (*P* < 0.05) (Figure 3(b)). Regarding the differential species at time 2, the species that increased in relative abundance were primarily members of the *Firmicutes* phylum, whereas the species that decreased in relative abundance were primarily members of the *Verrucomicrobia* phylum (*Supplementary 2*). Compared with those of the normal group, the DESeq2 volcano plots of the BSA group at time 2 showed decreases in the relative abundances of *Phascolarctobacterium* and *CF231* and increases in the relative abundances of *Blautia*, *Rothia*, *Jeotgalicoccus*, *Streptococcus*, *Coprobacillus*, and *Enterococcus* (*Supplementary 2*) (Supplementary data of DESeq2 analysis, *Supplementary 1*). A Kruskal–Wallis test revealed increases in the relative abundances of *Coprobacillus*, *rc4_4*, *Ruminococcus*, and *Blautia* and decreases in the relative abundances of *CF231* and *Akkermansia* in the BSA group (Figure 3(b)) (Supplementary data of Kruskal–Wallis test, *Supplementary 1*). LEfSe identified characteristic species in the BSA and normal group (*Supplementary 2*).

### 3.6. Correlations between the Gut Microbiome and BSA-Induced Liver Fibrosis

A significantly negative correlation was found between *CF231*, *Akkermansia*, and TBIL (*P* < 0.05) (Figure 3(d)). A significantly positive correlation was found between *Coprobacillus* and *Ruminococcus* and the AST/ALT (*P* < 0.05) (Figure 3(d)). A significantly positive correlation was found between *Streptococcus*, *Enterococcus*, *Ruminococcus*, *Coprobacillus*, *Jeotgalicoccus*, and *Blautia* and inflammatory factors (e.g., TNF-*α*, IL-1*β*, and IL-6), LN, Col-IV, and the thymus coefficient (*Supplementary 2*).

### 3.7. Evaluation of the Predictive Performance of the Gut Microbiome after BSA Induction

In terms of KEGG level 2 functions: in the gut microbiota of the BSA group, the expression of genes associated with biosynthesis of other secondary metabolites was downregulated and the expression of genes associated with infectious diseases (bacterial), metabolism of other amino acids and drug resistance (antimicrobial) was upregulated (*Supplementary 2*).

In terms of KEGG level 3 functions: in the gut microbiota of the BSA group, valine, leucine, and isoleucine biosynthesis; the peroxisome proliferators-activated receptors signaling pathway; biosynthesis of vancomycin group antibiotics; streptomycin biosynthesis; biosynthesis of amino acids; and biosynthesis of secondary metabolites was downregulated. In contrast, the expression of genes associated with D-alanine metabolism, ether lipid metabolism, purine metabolism, *Staphylococcus aureus* infection, cationic antimicrobial peptide resistance, arginine and proline metabolism, amino sugar and nucleotide sugar metabolism, and propanoate metabolism was upregulated (Figure 5(d)).

### 3.8. Serous Metabolites in BSA-Induced Liver Fibrosis Rats

The UHPLC-MS/MS analysis of serum samples revealed 17 differential metabolites in positive-ion mode (*Supplementary 2* and *Supplementary 1*) and 25 differential metabolites in negative-ion mode (*Supplementary 2* and *Supplementary 1*).

### 3.9. Correlations between Serous Metabolism and the Gut Microbiome in BSA-Induced Liver Fibrosis Rats

At time 2 in UHPLC-MS/MS positive-ion mode analyses, a positive correlation was found between *Rothia*, *Streptococcus*, *Enterococcus*, *Blautia*, *Jeotgalicoccus*, *Coprobacillus*, *Ruminococcus*, and deoxycytidine, choline, 56-dihydrothymine, cytosine, and cytidine (*Supplementary 2*). In negative-ion mode analyses, a positive correlation was found between *Akkermansia* and L-glutamic acid, gamma-aminobutyric acid (GABA), and *p*-ethylphenol, whereas a significant negative correlation was found between *Akkermansia* and pyrogallic acid (*P* < 0.05) (*Supplementary 2*). A significant positive correlation was found between *Akkermansia*, *Bifidobacterium*, and GABA (*P* < 0.05) (*Supplementary 2*). A significant negative correlation was found between *Coprobacillus* and beta-muricholate, melatonin, PAGln, 3-phenylpyruvic acid, isoferulic acid, glutathione, adenylic acid, chenodeoxycholate, and cholic acid (*P* < 0.05) (*Supplementary 2*). A positive correlation was found between *Bacteroides* and PAGln (*P* < 0.05), and a significant negative correlation was found between *Coprobacillus* and PAGln (*P* < 0.01) (*Supplementary 2*).

### 3.10. Evaluation of the Predictive Performance of the Gut Microbiome and Serous Metabolites in BSA-Induced Liver Fibrosis Rats

**Figure 6:**
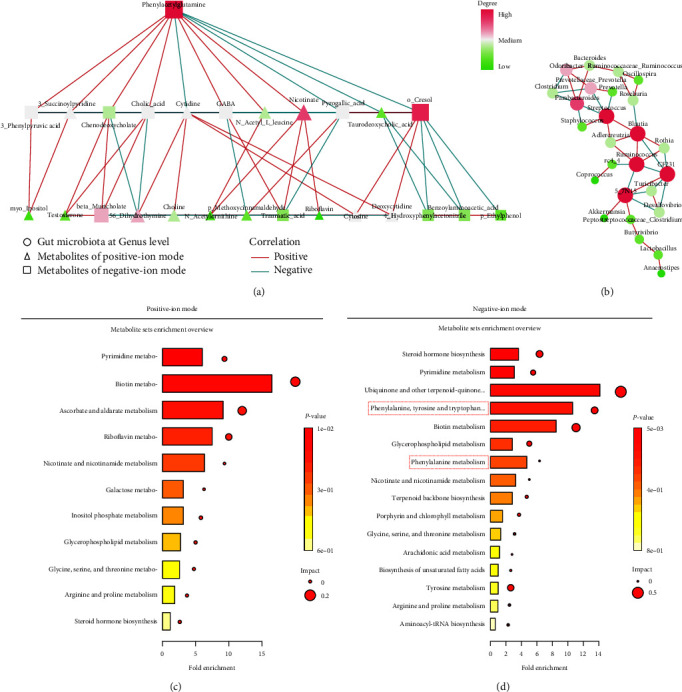
Spearman rank correlation network diagram ((a) and (b)). Pathway enrichment analysis (KEGG level 2) of the selected annotated differential metabolites was conducted. An abscissa represents multiple enrichment, whereas the size of a circle represents the number of factors enriched in the metabolic pathway ((c) and (d)).

Network analysis showed that PAGln was the most important serum metabolite in the BSA group (Figure 6(a)); a significant positive correlation was found between PAGln and 3-succinoylpyridine, 3-phenylpyruvic acid, cholic acid, GABA, *N*-acetyl-L-leucine, nicotinate, and *o*-cresol (Figure 6(a)). A positive correlation was found between *o*-cresol and deoxycytidine and cytosine (Figure 6(a)). A negative correlation was found between *o*-cresol and 4-hydroxyphenylacetonitrile, benzoylaminoacetic acid, and *p*-ethylphenol (*P* < 0.05) (Figure 6(a)). A negative correlation was found between *Streptococcus*, *Blautia*, *Ruminococcus*, *CF231*, and *5–7N15* (*P* < 0.05) (Figure 6(b)).

The KEGG level 2 functions of the metabolites in UHPLC-MS/MS positive-ion mode analyses were primarily associated with biotin metabolism, pyrimidine metabolism, ascorbate and aldarate metabolism, riboflavin metabolism, and nicotinate and nicotinamide metabolism (Figure 6(c)). The KEGG level 2 functions of the metabolites in the negative-ion mode UHPLC-MS/MS analyses were primarily associated with ubiquinone and other terpenoid-quinone metabolism; phenylalanine, tyrosine, and tryptophan metabolism; phenylalanine metabolism; biotin metabolism; steroid hormone biosynthesis; pyrimidine metabolism; and tyrosine metabolism (Figure 6(d)).

## 4. Discussion

In the BSA group, we observed stable and unambiguous histological changes in the liver tissue morphology and remarkably high concentrations of serum inflammatory biomarkers. After sensitization to BSA (a soluble antigen), B cells are activated to proliferate, differentiate, and mature, which results in their secreting anti-BSA antibodies into circulating blood. Thus, after tail-vein injection with BSA, B cells combine with anti-BSA antibodies to produce an IC comprising BSA and anti-BSA antibodies (a BSA-anti-BSA IC) [19, 23]. Furthermore, most of the IC in circulation rapidly fuses with erythrocytes by binding the C3b receptor. Subsequently, it appears that IC-bearing erythrocytes circulate through various organs, deposit most of their IC in the liver or spleen, and then return to the circulation [24]. At time 1, our data showed there were markedly elevated spleen coefficients in the BSA group, perhaps due to excessive retention of the IC caused by the increase of splenic blood flow [17]. Hepatic sinusoids have a network structure, and blood easily forms eddy currents, which increases IC retention in the liver. Continuous IC retention results in the activation of peripheral immune cells or HSCs, which causes progressive and chronic formation of inflammatory lesions. This may eventually lead to hepatocyte necrosis, fibroblast proliferation, and ECM protein proliferation [20]. In addition, an intravenous injection of cationic BSA can rapidly generate significant albuminuria and can be used to create an in vivo experimental animal model for investigating the pathophysiology of IC glomerulonephritis and for drug screening [25].

Our time 1 and 2 data showed no significant regression of the ECM in the BSA group. It is worth noting that the pathological scores of BSA group, such as IHC evaluation (Col-III and *α*-SMA), have decreased to some extent. This is probably due to the termination of BSA intervention, that is, the pathogenic factors equivalent to human beings have been removed, leading to a certain degree of reversal of liver fibrosis. This is also a normal phenomenon in the progress of liver fibrosis. Clinical and laboratory studies have found that myofibroblasts are inactivated after pathogen removal, which leads to the regression of liver fibrosis, indicating the reversibility of liver fibrosis [5]. In the progress of liver fibrosis, there is no clear time node to evaluate the deposition and regression of ECM [5]. Our results also support the above conclusions, indicating that BSA model can well simulate the progress of human liver fibrosis. In summary, our model had several important features: a high success rate, exemplary experimental reproducibility, and unambiguity in terms of histological changes in liver tissue morphology.

The gut microbiome is closely associated with liver diseases. On the one hand, a host provides nutrition and shelter to its gut microbiota, which help the host metabolize dietary nutrients or synthesize essential metabolites [26]. On the other hand, bile acid synthesis disorders can cause gut dysbiosis, increase gut permeability, cause inflammation, and alter the levels of gut microbiota metabolites, thereby promoting the development of liver disease [27, 28]. In research on diseases, interventions (such as drug treatment) are applied after the establishment of an animal model of a disease to determine how interventions affect the disease course [18, 29]. We wanted to use the BSA-induced liver fibrosis model to determine whether the gut microbiome was directly affected by BSA or was affected by BSA-induced liver fibrosis. Our data showed that the changes in the gut microbiota were more significant at time 1 than at time 2 and were accompanied by decreases in the relative abundances of characteristic species. It is worth noting that there are also complex vascular structures in the kidneys and intestines of the body. IC may also be deposited in these organs, which in turn affects organ function. Functional changes of multiple organs will affect intestinal homeostasis [26]. When the BSA intervention factors were removed, the functions of various organs gradually returned to normal, and the disorder degree of the affected intestinal flora also recovered. At time 2, the dysbiosis was primarily caused by BSA-induced liver fibrosis, which was the focus of the current study.

Our results revealed significant increases in the relative abundances of *Ruminococcus* and *Streptococcus* in the BSA-induced liver fibrosis rats. Moreover, a significant positive correlation was found between the relative abundances of *Streptococcus* and *Ruminococcus* and TNF-*α* in the BSA-induced liver fibrosis rats. These results corroborate those of previous studies. Specifically, *Ruminococcus* proliferated in a mouse bile drainage collection model [30] and may be an important member of the altered gut community in internal biliary drainage [31]. In addition, a significant increase in the abundance of *Ruminococcus* was seen in the gut microbiota of animals with nonalcoholic fatty liver disease [32]. *Streptococcus* may play an important role in the development of cirrhosis. Consistent with the results of the current study, the abundance of four *Streptococcus* strains was significantly increased in the gut microbiota of patients with liver cirrhosis [33]. The significant decrease in the abundance of *Akkermansia* we observed is also in agreement with the previous studies [34, 35]. *A. muciniphila* is a next-generation probiotic that can alleviate metabolic disorders and plays an important role in maintaining intestinal immune homeostasis in animals with alcoholic liver disease and nonalcoholic fatty liver disease [34, 35]. In addition, our data showed there was a significant negative correlation between *Akkermansia* and TBIL in BSA-induced liver fibrosis rats. However, we observed a significant increase in the abundance of *Blautia* in these rats, which disagrees with the results of previous studies; these have focused on the probiotic effects of *Blautia*, such as its biological transformation capacity and its ability to regulate host health and alleviate metabolic syndromes [36].

Gut bacteria not only play an important role in digestion, immune activation, and enteroendocrine signaling pathway regulation, but also communicate with the central nervous system or the liver and pancreas via specific metabolic compounds they produce, such as GABA, bile acids, short-chain fatty acids, glutamate, dopamine, and norepinephrine [37]. GABA signaling has potential therapeutic effects against inflammatory injury and improves the growth of animals under stress, and GABA supplementation can repair injured liver tissues by altering hepatic mitochondrial metabolism and proinflammatory cytokine expression [38]. Several other studies [39, 40] have shown that GABA can reverse the inflammatory response. For example, a synthetic analog of GABA exhibited anti-inflammatory and antioxidant activity in mice [39], while mice pretreated with GABA did not develop abnormal immune reactivity [40].

An important finding of our study is that there was a significant positive correlation between *Akkermansia*, *Bifidobacterium*, and GABA, with GABA expression reduced in the BSA-induced liver fibrosis rats. Gut microbiota that can decompose choline are obtained through the diet and produce trimethylamine *N*-oxide, and high concentrations of trimethylamine *N*-oxide in humans are associated with an increased risk of heart attack and stroke and variations in the concentrations of markers of renal function and inflammation [41]. Another important finding was that choline expression was increased in the BSA-induced liver fibrosis rats. Moreover, in UHPLC-MS/MS positive-ion mode analyses, a positive correlation was found between the relative abundances of *Rothia*, *Streptococcus*, *Enterococcus*, *Jeotgalicoccus*, *Coprobacillus*, *Ruminococcus*, and choline.

The most important finding was that phenylalanine/tyrosine metabolites (e.g., PAGln) were the predominant serum metabolites in the BSA-induced liver fibrosis rats, which was supported by the positive correlation between PAGln and GABA in our network analysis. Abnormal phenylalanine/tyrosine metabolism is involved in the occurrence and development of liver cancer [42]. Moreover, compared with patients without liver disease progression, patients with liver disease progress exhibit significantly alterations in phenylalanine, tyrosine, and tryptophan biosynthesis pathways and have increased serum concentrations of tyrosine [43]. Furthermore, pathway enrichment analyses showed that compared with healthy controls, patients with acute hepatitis E virus exhibit alterations in linoleic acid metabolism and in phenylalanine, tyrosine, and tryptophan biosynthesis [44]. Similarly, our pathway enrichment analysis showed increases in the expression of genes associated with phenylalanine, tyrosine, and tryptophan metabolism and with phenylalanine metabolism. Ursodeoxycholic acid, which is a metabolic by-product of intestinal bacteria, improves liver function via the phenylalanine/tyrosine pathway and induces microbiome remodeling in patients with liver dysfunction [45].

We also found a positive correlation between the relative abundance of *Bacteroides* and PAGln, and a significant negative correlation between the relative abundance *Coprobacillus* and PAGln. *Bacteroides acidifaciens* in the gut protects mice against liver injury [46], and *Coprobacillus* is positively correlated with tryptophan, an intestinal metabolite in mice [47]. In the absence of dietary fiber, *Coprobacillus* and *Lachnospiraceae* can enter the mucus layer of the intestine, leading to intestinal erosion, which damages intestinal integrity [48]. In terms of PAGln, research on cardiovascular diseases suggested that *porA* in microbes facilitates the conversion of dietary phenylalanine into phenylacetic acid, which is converted by the host to PAGln and then phenylacetylglycine, which fosters platelet responsiveness and thrombosis potential [49]. In summary, our results corroborate those of recent studies suggesting that phenylalanine and tyrosine metabolites play an important role in the association between gut microbiota and immune- and liver disease-related metabolites in serum.

## 5. Conclusion

Our BSA-induced liver fibrosis rat model exhibited several advantages: a good success rate, exemplary experimental reproducibility, relative stability, and unambiguous histological changes in the liver. This enabled us to explore gut microbiota disorder in BSA-induced liver fibrosis rats. We found that compared with the normal group, the BSA rats exhibited a significant decrease in the relative abundance of the beneficial bacteria *Akkermansia* and a significant increase in the relative abundance of the potentially pathogenic bacteria *Coprobacillus*, *Ruminococcus*, *Rothia*, *Streptococcus*, and *Blautia*. BSA-induced liver fibrosis can alter the metabolism of amino acids in the gut microbiota, especially that of phenylalanine and tyrosine, and thus can affect the generation of PAGln. This may be one of the reasons for the gut dysbiosis observed in the BSA-induced liver fibrosis rats.

## Data Availability

The datasets presented in this study can be found in online repositories. The names of the repository/repositories and accession number(s) are as follows: National Center for Biotechnology Information (NCBI) BioProject ID: PRJNA906122, https://submit.ncbi.nlm.nih.gov/subs/bioproject/.

## Abbreviations

BSA: Bovine serum albumin
HBV: Hepatitis B
HCV: Hepatitis C
HSCs: Hepatic stellate cells
ECM: Extracellular matrix
ELISA: Enzyme-linked immunosorbent assay
IL: Interleukin
TNF-*α*: Tumor Necrosis Factor Alpha
Col-IV: Type IV Collagen
LN: Laminin
H&E: Hematoxylin–eosin
*α*-SMA: *α*-smooth muscle actin
Col-I: Collagen I
Col-III: Collagen III
ALT: Serum alanine transaminase
AST: Aspartate aminotransferase
ALB: Albumin
TBIL: Total bilirubin
TP: Total protein
UHPLC-MS/MS: Ultrahigh-performance liquid chromatography-tandem mass spectrometry
LDA: Linear discriminant analysis
VIP: Variable importance in projection.

## Supplementary Materials

Supplementary 1 Table S1: list of differential metabolites in positive ion model. Table S2: list of differential metabolites in negative ion model. Table S3: the animal modeling rate. Table S4: Time 1 DESeq2 analysis data. Table S5: Time 2 DESeq2 analysis data. Table S6: Time 1 Kruskal–Wallis test data. Table S7: Time 2 Kruskal–Wallis test data.

Supplementary 2 Figure S1: expression of Col-I and Col-III in the liver tissue were detected through immunohistochemistry, and indicators of liver function are described. Figure S2: Venn diagram for BSA group-specific ASVs and those present in both groups. PCoA analysis was used to indicate the *β* diversity. The species distribution of the gut microbiota was described at the genus level. Figure S3: distribution of the characteristic species, DESeq2 analysis and LEfSe analyse. Figure S4: a genus-level phylogenetic tree with a heat map based on all the ASV data. Pathway enrichment analysis, and Spearman rank correlations were determined to assess the correlations between the significant gut microbiota species and environmental factors. Figure S5: differential metabolites in positive and negative ion model. Figure S6: a Spearman rank correlation was determined to assess the correlation between gut microbiota species and serum differential metabolites detected in positive-ion mode and negative-ion mode UHPLC-MS/MS analyses.
