# Supplementary material for: Alterations in the “Gut–Liver Axis” on Rats with Immunological Hepatic Fibrosis
Source: J Immunol Res. 2023 Sep 21;2023:5577850. doi: 10.1155/2023/5577850 (PMC10539088; doi:10.1155/2023/5577850)
Supplement: Supplementary 1 — Table S1: list of differential metabolites in positive ion model. Table S2: list of differential metabolites in negative ion model. Table S3: the animal modeling rate. Table S4: Time 1 DESeq2 analysis data. Table S5: Time 2 DESeq2 analysis data. Table S6: Time 1 Kruskal–Wallis test data. Table S7: Time 2 Kruskal–Wallis test data. [file 5577850.f1.doc]

**Supplemental Material Tables**

**Table.S1 List of differential metabolites in positive ion model.**

| Namber | Metabolites | p-value | FC | VIP | normal-Mean | BSA-Mean | Change |
| --- | --- | --- | --- | --- | --- | --- | --- |
| 1 | Choline | 0.0329 | 1.2528 | 1.3876 | 76.6161 | 95.9808 | ↑ |
| 2 | Cytosine | 0.0004 | 1.4982 | 2.1142 | 15.8463 | 23.7406 | ↑ |
| 3 | Cytidine | ＜0.0001 | 1.6264 | 2.4703 | 2.2550 | 3.6675 | ↑ |
| 4 | Deoxycytidine | 0.0290 | 1.2202 | 1.3291 | 11.7476 | 14.3341 | ↑ |
| 5 | 5,6-Dihydrothymine | 0.0073 | 1.7928 | 2.0733 | 8.4008 | 15.0609 | ↑ |
| 6 | Taurodeoxycholate | 0.0102 | 3.3739 | 1.8660 | 0.1881 | 0.6345 | ↑ |
| 7 | p-Methoxycinnamaldehyde | 0.0257 | 1.3693 | 1.4428 | 1.1156 | 1.5276 | ↑ |
| 8 | Biotin | 0.0037 | -0.5951 | 1.8806 | 0.3369 | 0.2005 | ↓ |
| 9 | myo-Inositol | 0.0333 | -0.8263 | 1.6170 | 1260.7883 | 1041.8259 | ↓ |
| 10 | Nicotinate | 0.0032 | -0.7754 | 1.8621 | 5.3311 | 4.1337 | ↓ |
| 11 | Riboflavin | 0.0052 | -0.7345 | 2.2967 | 1.0989 | 0.8071 | ↓ |
| 12 | N-Acetylornithine | 0.0471 | -0.8068 | 1.2596 | 3.5317 | 2.8495 | ↓ |
| 13 | Testosterone | 0.0046 | -0.4952 | 1.9834 | 0.7293 | 0.3612 | ↓ |
| 14 | N-Acetyl-L-leucine | 0.0384 | -0.6093 | 1.5588 | 2.5122 | 1.5308 | ↓ |
| 15 | Isoproterenol | 0.0183 | -0.6143 | 1.8906 | 0.1263 | 0.0776 | ↓ |
| 16 | Daidzein | ＜0.0001 | -0.1722 | 2.7849 | 0.5679 | 0.0978 | ↓ |
| 17 | 3-Succinoylpyridine | 0.0081 | -0.7056 | 1.7369 | 2.2566 | 1.5923 | ↓ |

**Table.S2 List of differential metabolites in negative ion model.**

| Namber | Metabolites | p-value | FC | VIP | normal-Mean | BSA-Mean | Change |
| --- | --- | --- | --- | --- | --- | --- | --- |
| 1 | 3alpha-Hydroxy-5alpha-androstan-17-one | 0.0473 | 1.4227 | 1.1356 | 0.1345 | 0.0813 | ↑ |
| 2 | o-Cresol | 0.0021 | 4.1633 | 1.4471 | 117.9783 | 54.4679 | ↑ |
| 3 | Pyrogallic acid | 0.0005 | 1.9182 | 1.7102 | 8.2928 | 5.7761 | ↑ |
| 4 | Taurolithocholic acid | 0.0337 | -0.2269 | 1.3845 | 24.4794 | 19.4981 | ↓ |
| 5 | Syringic acid | 0.0294 | -0.7187 | 1.2696 | 58.3883 | 49.1252 | ↓ |
| 6 | Adenylic acid | 0.0281 | -0.6044 | 1.2363 | 0.6131 | 0.3031 | ↓ |
| 7 | 3-Phenylpyruvic acid | 0.0264 | -0.8296 | 1.0783 | 6.1411 | 5.0945 | ↓ |
| 8 | beta-Muricholate | 0.0244 | -0.5748 | 1.2528 | 81.7014 | 122.3603 | ↓ |
| 9 | Traumatic acid | 0.0225 | -0.7759 | 1.1177 | 0.6184 | 0.4775 | ↓ |
| 10 | Suberic acid | 0.0222 | -0.6965 | 1.2780 | 0.0901 | 0.1281 | ↓ |
| 11 | L-Glutamic aci | 0.0169 | -0.7965 | 1.2018 | 0.4588 | 0.2562 | ↓ |
| 12 | alpha-Ketoglutaric acid | 0.0161 | -0.8414 | 1.1460 | 0.5005 | 0.9600 | ↓ |
| 13 | Icosanoic acid | 0.0084 | -0.7117 | 1.2953 | 0.4138 | 1.7227 | ↓ |
| 14 | 4-Hydroxy-2-quinolinecarboxylic acid | 0.0073 | -0.7752 | 1.2128 | 119.1899 | 54.8568 | ↓ |
| 15 | Glutathione | 0.0068 | -0.4944 | 1.4873 | 0.1310 | 0.0435 | ↓ |
| 16 | Phenylacetylglutamine | 0.0049 | -0.5063 | 1.6073 | 0.9150 | 0.7093 | ↓ |
| 17 | GABA | 0.0041 | -0.7721 | 1.6011 | 2.4266 | 0.6680 | ↓ |

**Table2 (continued)**

| Namber | Metabolites | p-value | FC | VIP | normal-Mean | BSA-Mean | Change |
| --- | --- | --- | --- | --- | --- | --- | --- |
| 18 | Melatonin | 0.0022 | -0.3318 | 1.8552 | 0.2586 | 0.0587 | ↓ |
| 19 | Chenodeoxycholate | 0.0015 | -0.2753 | 1.7622 | 4.5389 | 2.2980 | ↓ |
| 20 | p-Ethylphenol | 0.0014 | -0.5280 | 1.4403 | 0.4661 | 0.3317 | ↓ |
| 21 | Cholic acid | 0.0005 | -0.3508 | 1.7358 | 0.7319 | 0.3049 | ↓ |
| 22 | Isoferulic acid | ＜0.0001 | -0.4165 | 1.8356 | 0.0581 | 0.0418 | ↓ |
| 23 | Benzoylaminoacetic acid | ＜0.0001 | -0.4603 | 1.7978 | 8.0584 | 4.2546 | ↓ |
| 24 | 4-Hydroxyphenylacetonitrile | ＜0.0001 | -0.4617 | 1.7982 | 0.6539 | 0.5074 | ↓ |
| 25 | PGD2 | ＜0.0001 | -0.5584 | 1.9617 | 16.7954 | 9.6531 | ↓ |

**Table.S3 The animal modeling rate.**

| Category | Positive of anti-BSA antibodies | mortality | liver fibrosis at Time1* | liver fibrosis at Time2* |
| --- | --- | --- | --- | --- |
| Normal | 0.00% | 0.00% | 0.00% | 0.00% |
| BSA | 90.00% | 17.86%^#^ | 100.00% | 100.00% |

Table.S3. Notes: Given that appetite and weight loss may occur when animals suffer from liver disease, the criteria for humane endpoints in the experiment were as follows: the rats ate significantly less than they had done or their body weight decreased rapidly by >10%, or the rats were in a poor mental state for prolonged period and did not gain body weight. If any of the above conditions were observed, the rats were euthanized to relieve their suffering. #An animal was euthanized, which was not included in the mortality calculations. * Liver fibrosis was determined by positive H&E and Sirius Red staining and by immunohistochemical pathological examinations for Col-I and Col-III in sections.

**Table.S4 Time1 DESeq2 analysis data.**

| ASVs | Base Mean | log2 Fold Change | lfcSE | stat | p-value | p-adj |
| --- | --- | --- | --- | --- | --- | --- |
| Prevotellaceae_Prevotella | 329.2146 | 2.3795 | 0.6082 | 3.9124 | 0.0001 | 0.0037 |
| Sutterella | 166.5859 | -1.5475 | 0.4317 | -3.5847 | 0.0003 | 0.0069 |
| Lactobacillus | 3472.0749 | -1.6255 | 0.5134 | -3.1663 | 0.0015 | 0.0211 |
| Anaerostipes | 22.7648 | 1.6624 | 0.5463 | 3.0427 | 0.0023 | 0.0240 |
| rc4_4 | 374.8864 | -1.9216 | 0.6534 | -2.9409 | 0.0033 | 0.0268 |
| Corynebacterium | 4.7721 | 2.4443 | 0.8970 | 2.7249 | 0.0064 | 0.0303 |
| Roseburia | 386.6023 | 1.2795 | 0.4637 | 2.7596 | 0.0058 | 0.0303 |
| Halomonas | 17.1958 | 1.9832 | 0.7025 | 2.8230 | 0.0048 | 0.0303 |
| Akkermansia | 31.9654 | -2.9791 | 1.0978 | -2.7136 | 0.0067 | 0.0303 |
| Bacillus | 14.8512 | 2.2445 | 0.8727 | 2.5719 | 0.0101 | 0.0415 |
| Turicibacter | 1411.8831 | 0.9242 | 0.3840 | 2.4065 | 0.0161 | 0.0600 |
| Odoribacter | 46.3603 | -0.9802 | 0.4152 | -2.3610 | 0.0182 | 0.0623 |
| Erysipelotrichaceae_Clostridium | 4.9217 | -1.7379 | 0.7768 | -2.2372 | 0.0253 | 0.0797 |
| Desulfovibrio | 45.3038 | -1.2924 | 0.6086 | -2.1236 | 0.0337 | 0.0987 |
| Dietzia | 9.1891 | 1.7171 | 0.8724 | 1.9684 | 0.0490 | 0.1306 |

Table.S4. Notes: Base Mean represents the average abundance of all samples, and log2 Fold Change corresponds to log2 (normal group/ BSA group). Log2 Fold Change greater than zero means that the abundance of normal group is greater than that of BSA group. LfcSE corresponds to the standard error of log2 Fold Change. Stat corresponds to test statistics, p-value and p-adj correspond to P value and corrected P value respectively.

**Table.S5 Time2 DESeq2 analysis data.**

| ASVs | Base Mean | log2 Fold Change | lfcSE | stat | p-value | p-adj |
| --- | --- | --- | --- | --- | --- | --- |
| Blautia | 24.1368 | -5.5342 | 1.0700 | -5.1721 | ≤0.001 | ≤0.001 |
| Rothia | 62.2735 | -4.7248 | 0.9393 | -5.0299 | ≤0.001 | ≤0.001 |
| Jeotgalicoccus | 6.9977 | -3.6650 | 1.0277 | -3.5662 | 0.0004 | 0.0057 |
| Streptococcus | 39.8399 | -3.3312 | 1.0361 | -3.2153 | 0.0013 | 0.0153 |
| Coprobacillus | 8.6434 | -2.4984 | 0.8111 | -3.0802 | 0.0021 | 0.0194 |
| Adlercreutzia | 273.0412 | -1.8060 | 0.6553 | -2.7561 | 0.0058 | 0.0393 |
| CF231 | 21.0863 | 2.4620 | 0.8861 | 2.7785 | 0.0055 | 0.0393 |
| Enterococcus | 9.6909 | -2.6235 | 0.9705 | -2.7033 | 0.0069 | 0.0403 |
| Staphylococcus | 8.1221 | -2.7584 | 1.0773 | -2.5605 | 0.0105 | 0.0546 |
| Ruminococcus | 216.4080 | -1.4108 | 0.5695 | -2.4773 | 0.0132 | 0.0622 |
| Phascolarctobacterium | 3.7005 | 2.4585 | 1.0286 | 2.3901 | 0.0168 | 0.0720 |
| Allobaculum | 6.0115 | -2.1540 | 0.9785 | -2.2013 | 0.0277 | 0.1085 |
| Akkermansia | 11.0325 | 1.7602 | 0.8190 | 2.1491 | 0.0316 | 0.1143 |
| Aggregatibacter | 5.4097 | -2.0050 | 0.9523 | -2.1054 | 0.0353 | 0.1184 |
| rc4_4 | 19.2221 | 1.5063 | 0.7272 | 2.0715 | 0.0383 | 0.1200 |

Table.S5. Notes: Base Mean represents the average abundance of all samples, and log2 Fold Change corresponds to log2 (normal group/ BSA group). Log2 Fold Change greater than zero means that the abundance of normal group is greater than that of BSA group. LfcSE corresponds to the standard error of log2 Fold Change. Stat corresponds to test statistics, p-value and p-adj correspond to P value and corrected P value respectively.

**Table.S6 Time1 Kruskal-Wallis test data.**

| ASVs | Test-Statistic | p-value | Control-mean | Model-mean |
| --- | --- | --- | --- | --- |
| Lactobacillus | 6.8182 | 0.0090 | 1559.0000 | 4631.4000 |
| Sutterella | 6.8182 | 0.0090 | 76.8000 | 232.6000 |
| Prevotellaceae_Prevotella | 5.7709 | 0.0163 | 530.8000 | 93.8000 |
| Anaerostipes | 5.3448 | 0.0208 | 30.0000 | 8.6000 |
| Desulfovibrio | 4.8109 | 0.0283 | 22.6000 | 57.4000 |
| Erysipelotrichaceae_Clostridium | 4.7059 | 0.0301 | 1.0000 | 5.8000 |
| Halomonas | 4.5283 | 0.0333 | 27.2000 | 5.4000 |
| Geobacter | 4.5125 | 0.0336 | 0.4000 | 3.4000 |
| Odoribacter | 3.9382 | 0.0472 | 27.4000 | 55.4000 |
| Turicibacter | 3.9382 | 0.0472 | 1714.4000 | 896.4000 |
| Coprococcus | 3.9382 | 0.0472 | 813.2000 | 445.6000 |
| Roseburia | 3.9382 | 0.0472 | 496.8000 | 198.2000 |
| Akkermansia | 0.7200 | 0.3961 | 5.2000 | 47.4000 |

Table.S6. Notes: Kruskal-Wallis test provide Test-Statistic, total P-value, and average values of each group.

**Table.S7 Time2 Kruskal-Wallis test data.**

| ASVs | Test-Statistic | p-value | Control-mean | Model-mean |
| --- | --- | --- | --- | --- |
| Ruminococcus | 6.8182 | 0.0090 | 114.8000 | 293.2000 |
| rc4_4 | 6.8182 | 0.0090 | 25.2000 | 8.2000 |
| Blautia | 5.5385 | 0.0186 | 0.0000 | 41.8000 |
| Coprobacillus | 5.4450 | 0.0196 | 1.4000 | 12.6000 |
| CF231 | 4.9613 | 0.0259 | 35.2000 | 4.8000 |
| Akkermansia | 3.9865 | 0.0459 | 16.4000 | 3.8000 |

Table.S7. Notes: Kruskal-Wallis test provide Test-Statistic, total P-value, and average values of each group.
