# Supplementary material for: Alterations in the “Gut–Liver Axis” on Rats with Immunological Hepatic Fibrosis
Source: J Immunol Res. 2023 Sep 21;2023:5577850. doi: 10.1155/2023/5577850 (PMC10539088; doi:10.1155/2023/5577850)
Supplement: Supplementary 2 — Figure S1: expression of Col-I and Col-III in the liver tissue were detected through immunohistochemistry, and indicators of liver function are described. Figure S2: Venn diagram for BSA group-specific ASVs and those present in both groups. PCoA analysis was used to indicate the β diversity. The species distribution of the gut microbiota was described at the genus level. Figure S3: distribution of the characteristic species, DESeq2 analysis and LEfSe analyse. Figure S4: a genus-level phylogenetic tree with a heat map based on all the ASV data. Pathway enrichment analysis, and Spearman rank correlations were determined to assess the correlations between the significant gut microbiota species and environmental factors. Figure S5: differential metabolites in positive and negative ion model. Figure S6: a Spearman rank correlation was determined to assess the correlation between gut microbiota species and serum differential metabolites detected in positive-ion mode and negative-ion mode UHPLC-MS/MS analyses. [file 5577850.f2.doc]

**Supplemental Material figures**


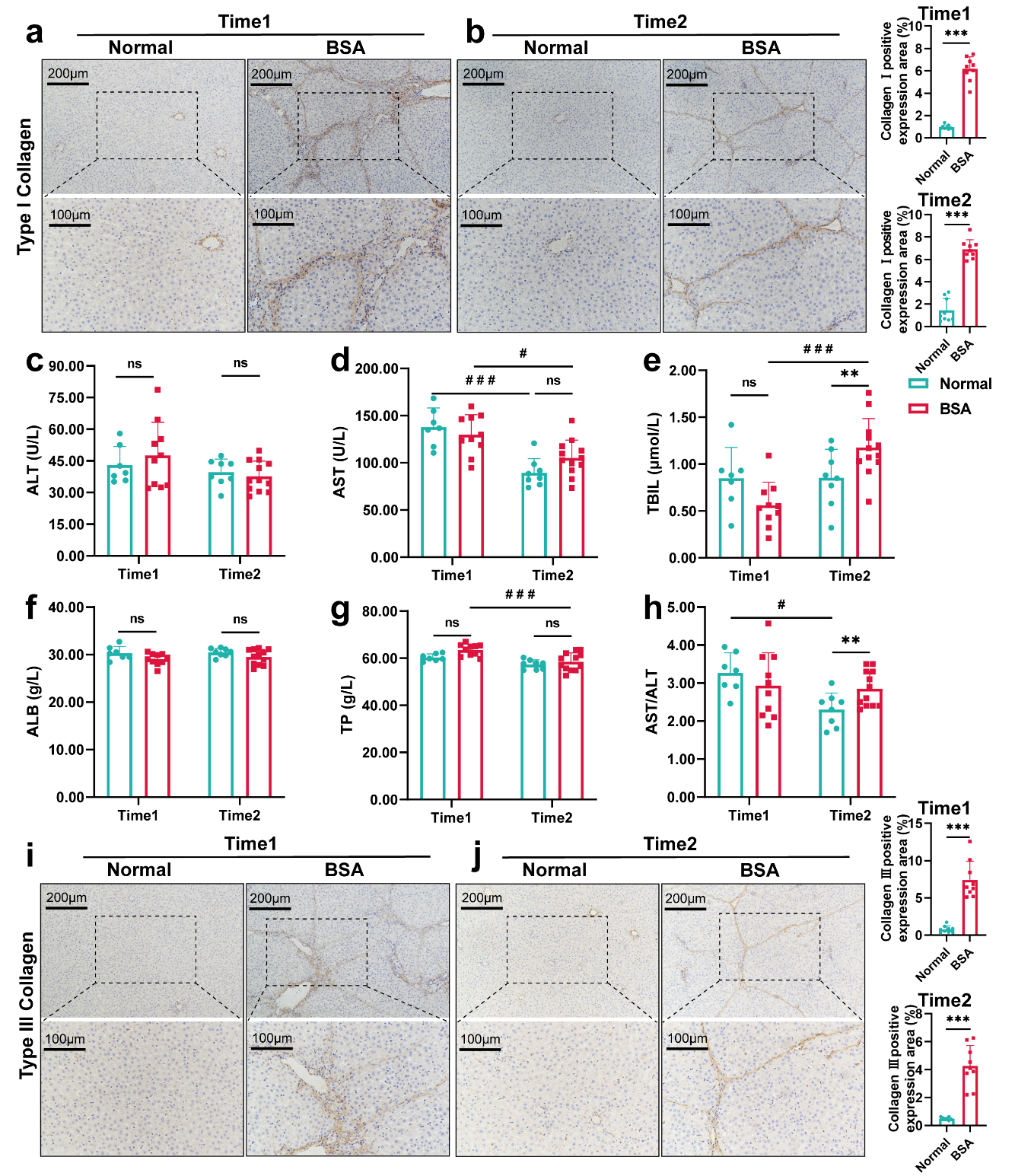
**Figure S1**

Fig. S1: Distribution and expression of Col-I (a and b) and Col-III (i and j) in the liver tissue were detected through immunohistochemistry. Indicators of liver function are described in the histograms (c, d, e, f, g, and h). Results compared with the normal group; *****P*** < 0.01. Results compared with time 1, #***P*** < 0.05 and ###***P*** < 0.001. NS indicates nonsignificance.


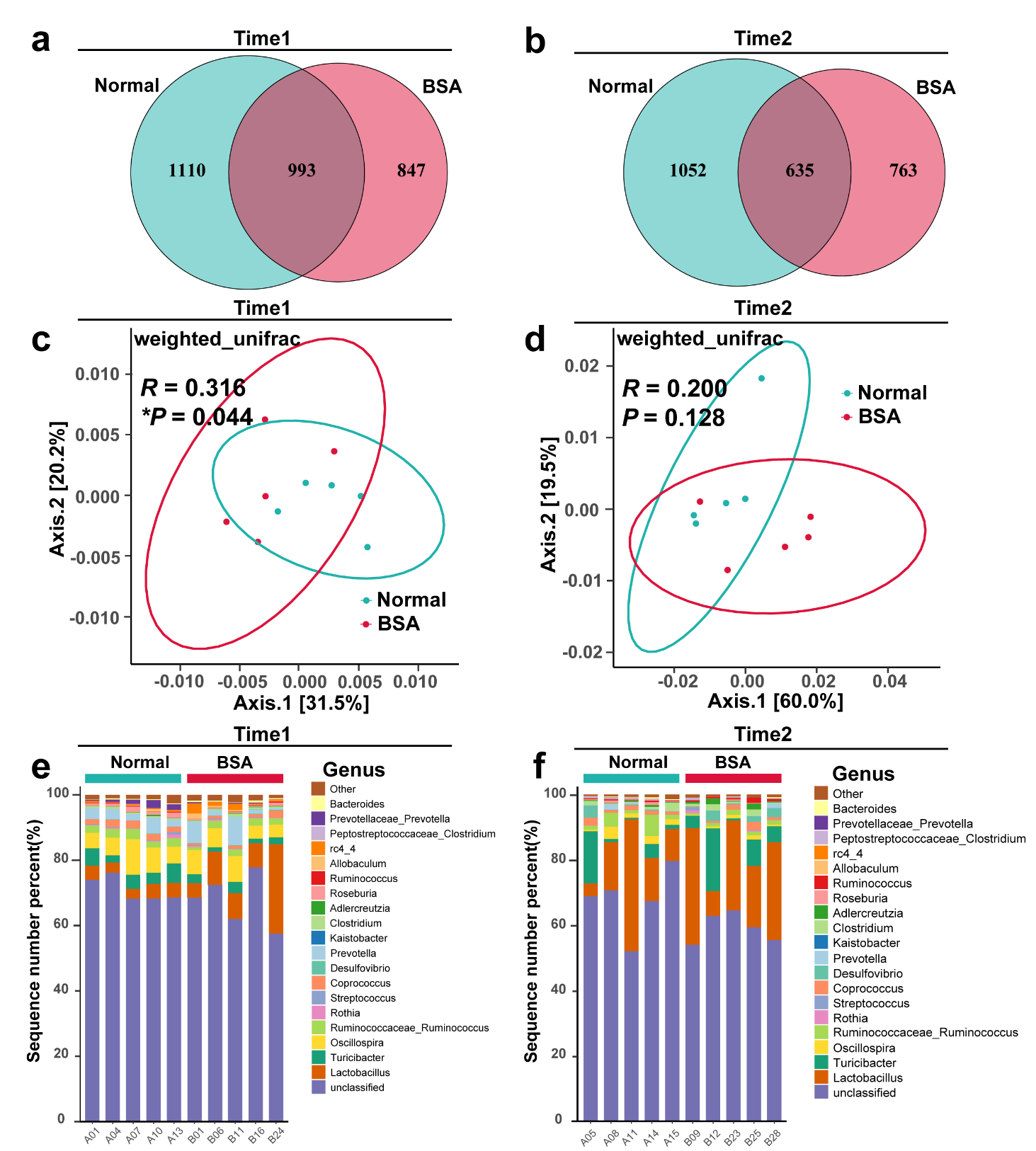
**Figure S2**

Fig. S2: Venn diagram for BSA group-specific ASVs and those present in both groups (a and b). The PCoA analysis (c and d). The species distribution of the gut microbiota was described at the genus level (e and f).


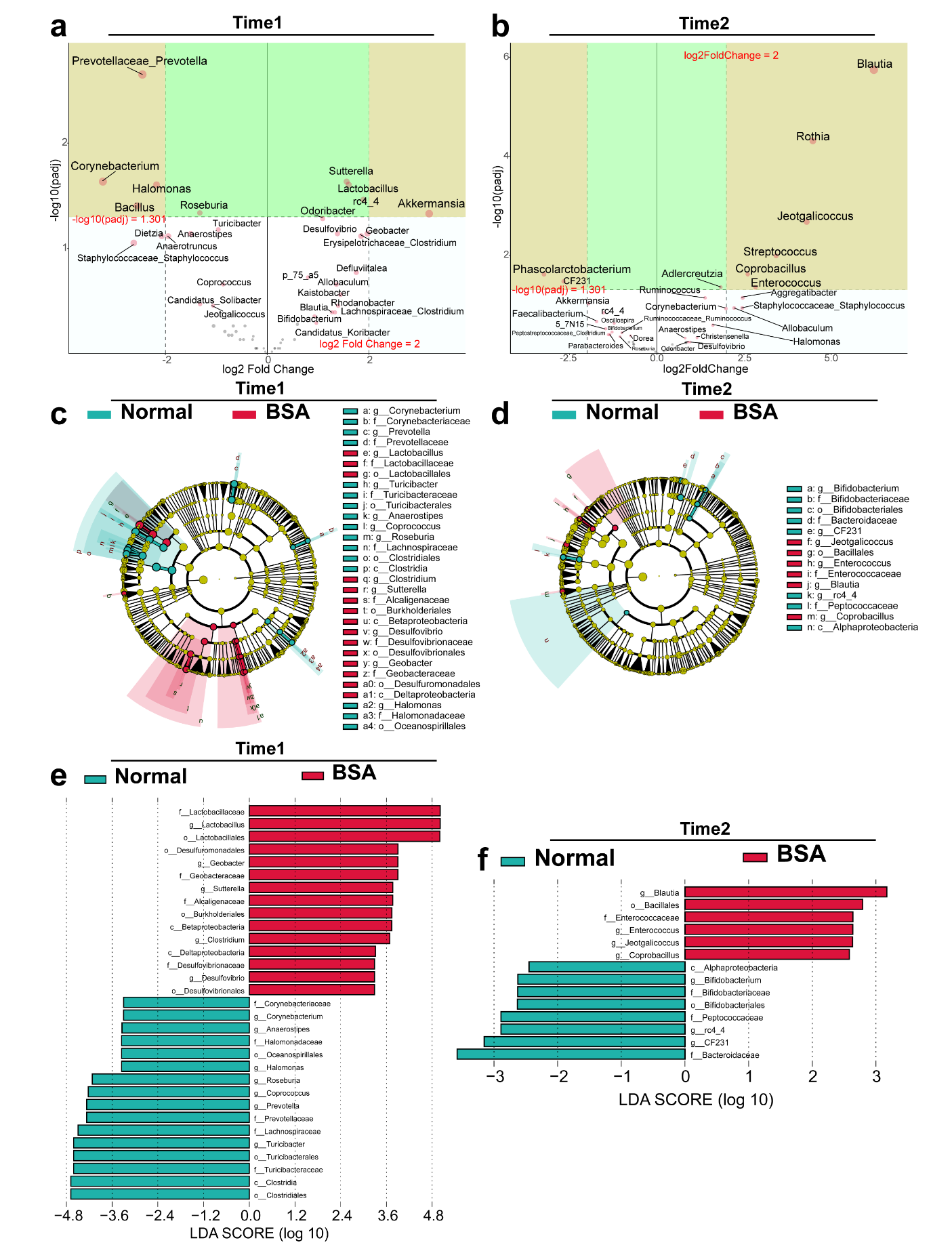
**Figure S3**

Fig. S3: DESeq2 analysis at time 1 and time 2 are shown in panels (a) and (b), respectively. The dots represent amplicon sequence variants (ASVs). The LEfSe analyse (c and d). The cladogram diagram of the species evolution branch corresponds to different levels of phyla, families, and genera, respectively, from inside to outside. Each node represents a species, and the nonsignificant species are colored entirely yellow. The significant species were assessed (e and f).


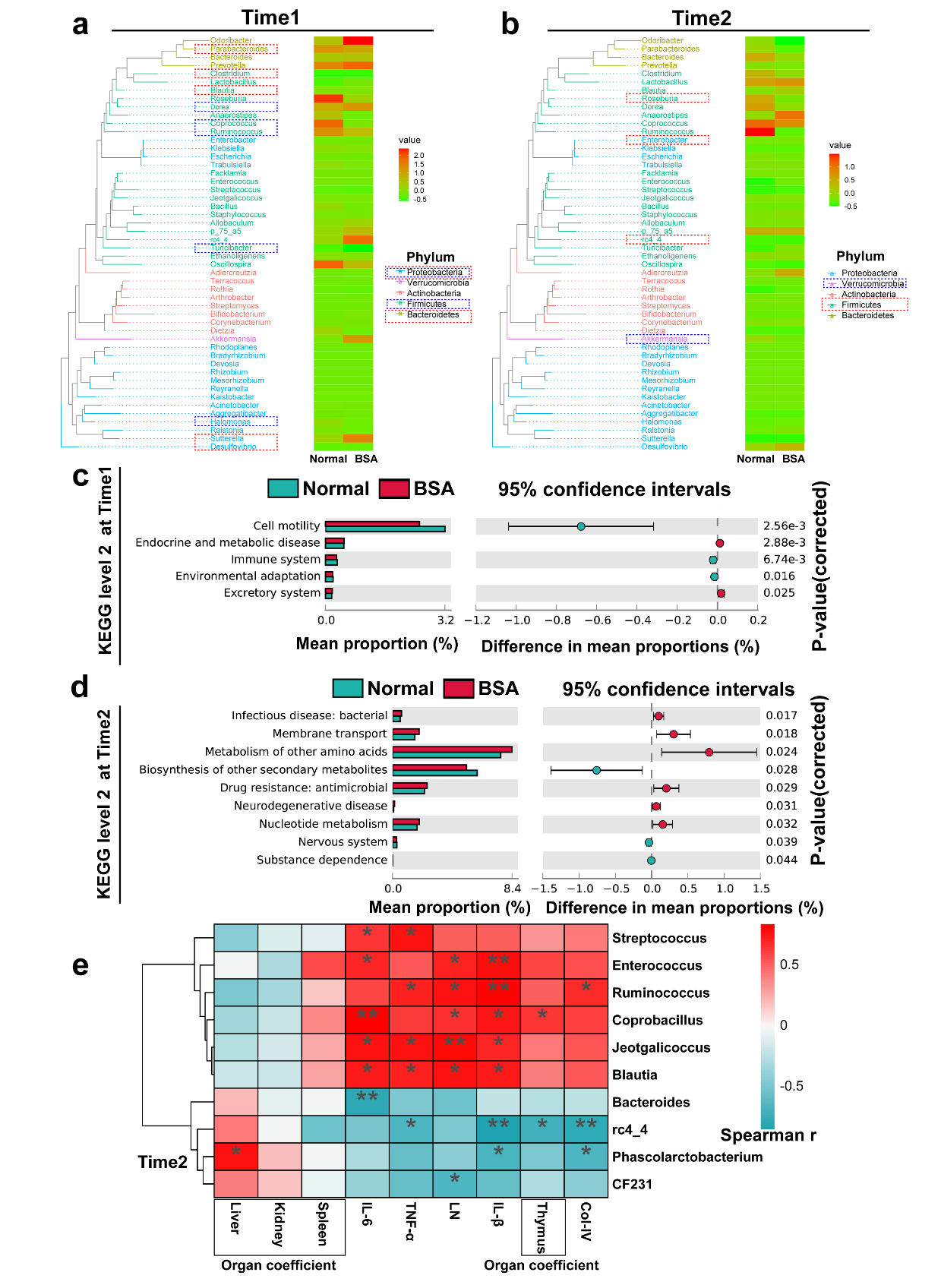
**Figure S4**

Fig. S4: A genus-level phylogenetic tree with a heat map based on all of the ASV data. The red dashed box represents an increase in relative abundance, whereas the blue dashed box represents a decrease in relative abundance (a and b). Pathway enrichment analysis (level 2) of selected annotated differential species (c and d). Spearman rank correlations (e). ****P*** < 0.05, *****P*** < 0.01, and ******P*** < 0.001.


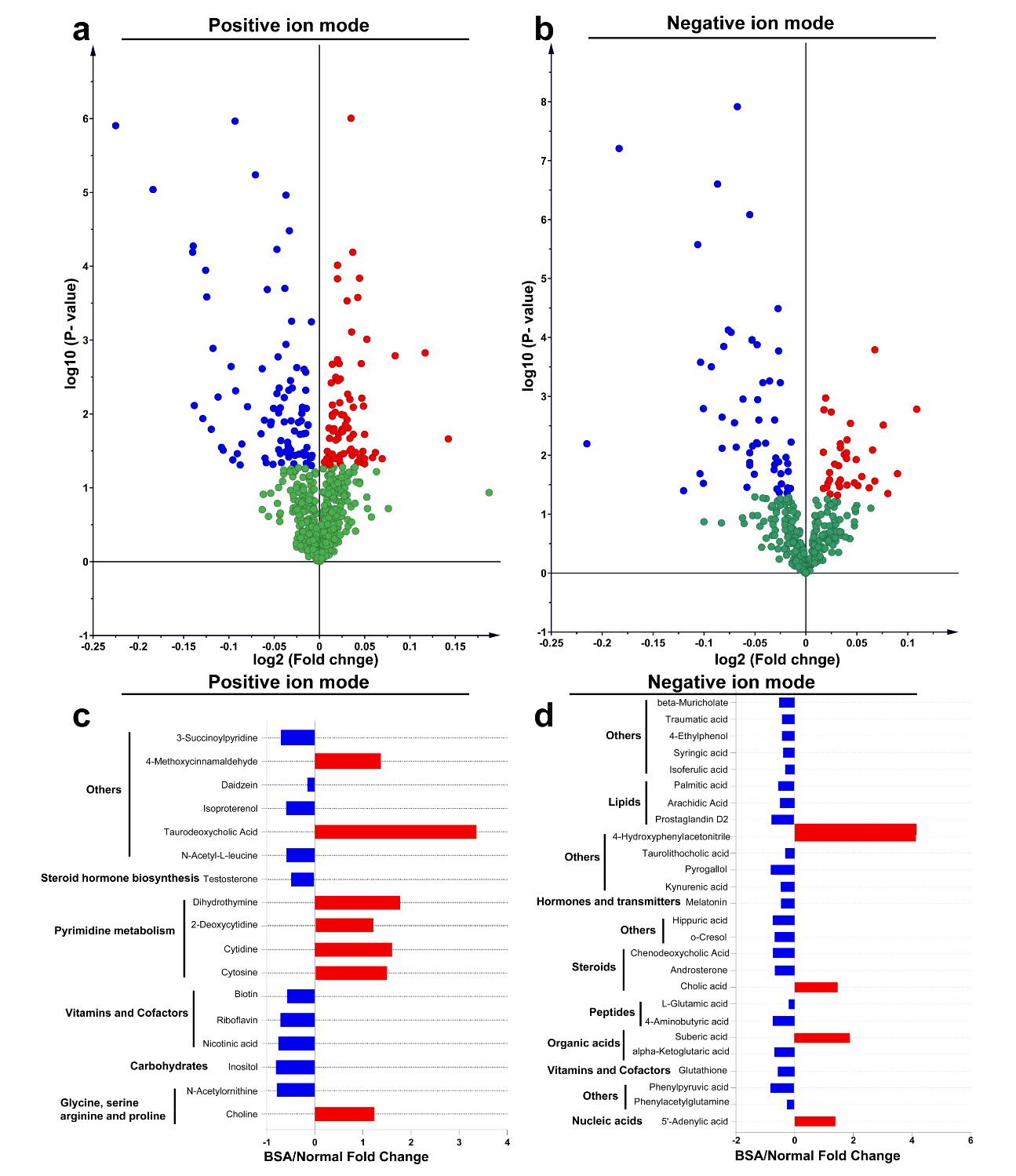
**Figure S5**

Fig. S5: For the BSA group and relative to the normal group, the red dots indicate upregulated metabolites and the blue dots indicate downregulated metabolites (a and b). For the BSA group and relative to the normal group, the red bar graph indicates upregulated metabolites, whereas the blue bar graph indicates downregulated metabolites (c and d).


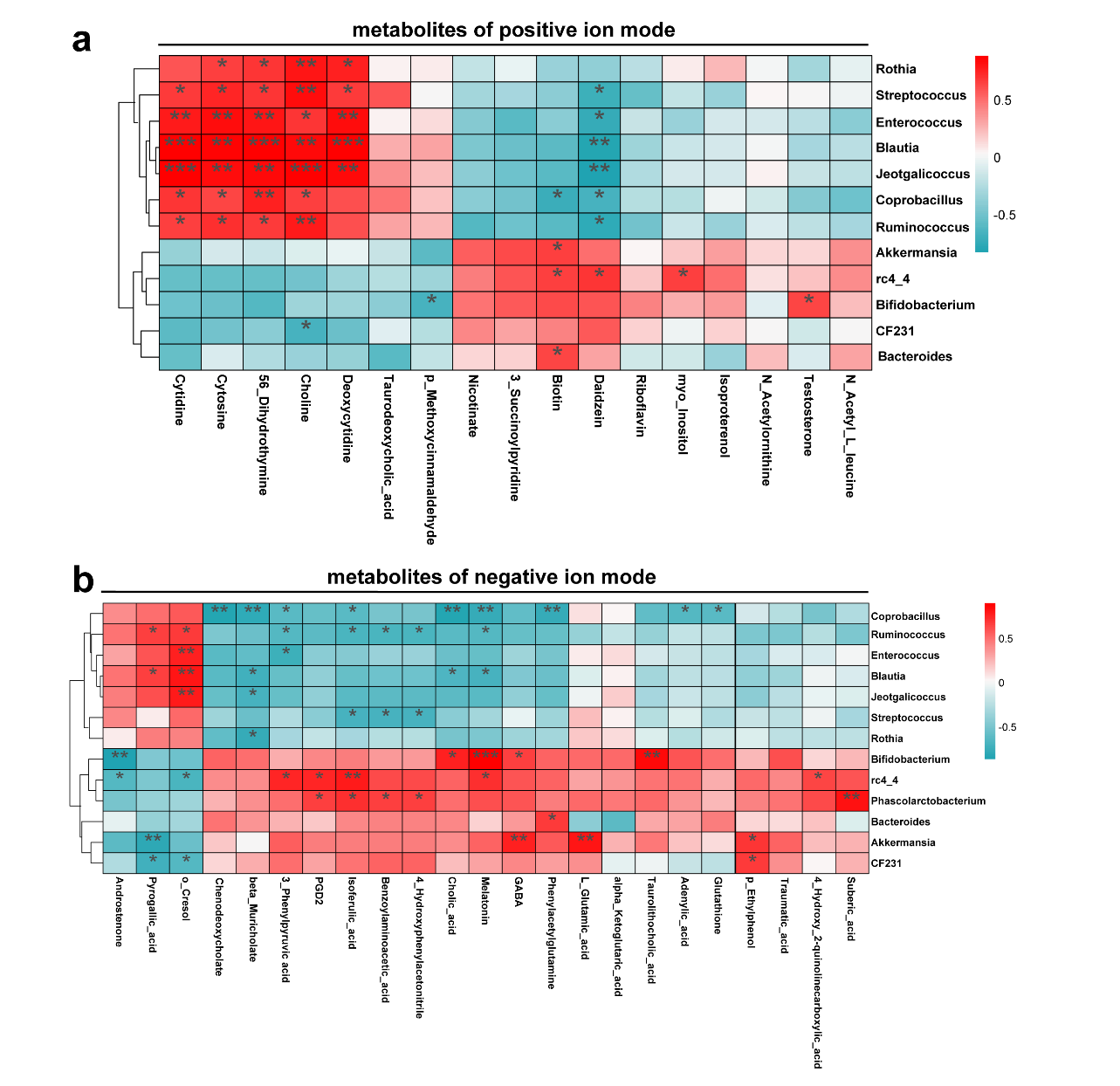
**Figure S6**

Fig. S6: A Spearman rank correlation (a and b). Compared with the normal group, ****P*** < 0.05, *****P*** < 0.01, and ******P*** < 0.001.
